# Spatial and Working Memory Is Linked to Spine Density and Mushroom Spines

**DOI:** 10.1371/journal.pone.0139739

**Published:** 2015-10-15

**Authors:** Rasha Refaat Mahmmoud, Sunetra Sase, Yogesh D. Aher, Ajinkya Sase, Marion Gröger, Maher Mokhtar, Harald Höger, Gert Lubec

**Affiliations:** 1 Department of Pharmaceutical Chemistry, University of Vienna, 1090 Vienna, Austria; 2 CF Imaging, Medical University of Vienna, 1090 Vienna, Austria; 3 Core Unit of Biomedical Research, Division of Laboratory Animal Science and Genetics, Medical University of Vienna, Brauhausgasse 34, A-2325 Himberg, Austria; 4 Department of Pediatrics, Faculty of Medicine, Assuit University, Assuit, Egypt; Université Pierre et Marie Curie, FRANCE

## Abstract

**Background:**

Changes in synaptic structure and efficacy including dendritic spine number and morphology have been shown to underlie neuronal activity and size. Moreover, the shapes of individual dendritic spines were proposed to correlate with their capacity for structural change. Spine numbers and morphology were reported to parallel memory formation in the rat using a water maze but, so far, there is no information on spine counts or shape in the radial arm maze (RAM), a frequently used paradigm for the evaluation of complex memory formation in the rodent.

**Methods:**

24 male Sprague-Dawley rats were divided into three groups, 8 were trained, 8 remained untrained in the RAM and 8 rats served as cage controls. Dendritic spine numbers and individual spine forms were counted in CA1, CA3 areas and dentate gyrus of hippocampus using a DIL dye method with subsequent quantification by the Neuronstudio software and the image J program.

**Results:**

Working memory errors (WME) and latency in the RAM were decreased along the training period indicating that animals performed the task. Total spine density was significantly increased following training in the RAM as compared to untrained rats and cage controls. The number of mushroom spines was significantly increased in the trained as compared to untrained and cage controls. Negative significant correlations between spine density and WME were observed in CA1 basal dendrites and in CA3 apical and basal dendrites. In addition, there was a significant negative correlation between spine density and latency in CA3 basal dendrites.

**Conclusion:**

The study shows that spine numbers are significantly increased in the trained group, an observation that may suggest the use of this method representing a morphological parameter for memory formation studies in the RAM. Herein, correlations between WME and latency in the RAM and spine density revealed a link between spine numbers and performance in the RAM.

## Introduction

Changes in synaptic structure that usually occur by activity have been proposed to underlie learning and memory. Dendritic spines, which are specialized protrusions that form the site for excitatory synaptic contact, can undergo changes in size, shape and number in response to activity. The dendritic spine density is a good marker for the number of hippocampal excitatory synapses. Since learning effects are long-lasting, structural changes that occur to hippocampal synapses are supposed to correlate with spatial learning **[1–3]**.

The hippocampal formation is closely related to spatial learning and memory. Many of the hippocampal synapses have plastic properties, which were proposed to play a vital role in the learning process **[4]**.The hippocampus plays an important role in the declarative form of memory, which means retrieving daily facts and incidences[5, 6]. In addition, recent work that was done on neuronal firing in animal and human behaviour, using functional imaging of brain in humans has demonstrated the importance of the hippocampus for spatial memory. Neuropsychological analyses of humans and animals that had hippocampal damage, revealed how hippocampal neurons can carry out elemental cognitive processes to perform declarative memory **[7]**.

A strong association of dendritic spine density in the hippocampus and memory has been established using several behavioural paradigms. The encoding of newly formed memories in a conditioning paradigm showed increased spine density in CA1 pyramidal cells in adult male rats [1, 8] and female rats [9]. It was shown that dendritic spine density was increased on pyramidal cells in CA1 following performance of two different spatial memory tasks, the Morris water maze and object placement which might suggest that they are morphological substrates for memory [3, 10]. Moreover, structural changes of hippocampal synapses and formation of new synaptic contacts has been proposed to be a possible mechanism in the late phase of long-term potentiation (LTP), a correlate of plasticity, which is involved in learning and memory[11]. In addition it was shown that existing spines in the hippocampus endure structural modifications that result in LTP [1, 8]. It has been illustrated that LTP induces increased spine density [12] while long-term depression reduces spine numbers[13]. In addition, new spines were formed in dentate gyrus following LTP [14]. This indicates that there is a strong connection between dendritic spine density and memory in the hippocampus.

The aforementioned data signifies that spatial memory performance results in increased spine density. However, the spine morphology following the performance in the radial arm maze has not been published so far. Herein, dendritic spine morphology, density, types and numbers of spines were examined in hippocampi of rats trained in the radial arm maze (RAM), a paradigm for testing spatial working memory (WM) and indeed, an increase of mushroom spine density in the trained group was observed along with a significant correlation between spine density and working memory errors and latency.

## Material and Methods

### 1. Radial arm maze (RAM)

#### 1.1 Animals

Male Sprague Dawley rats, aged between 12–14 weeks, were used in the experiment. They were bred and maintained in cages made of Makrolon and filled with autoclaved woodchips in the Core Unit of Biomedical Research, Division of Laboratory Animal Science and Genetics, Medical University of Vienna. Food and water in bottles was available ad libitum. The room was illuminated with artificial light at an intensity of about 200 lx in 2 m from 5 am to 7 pm. Experiments were carried out between 8 am and 2 pm. All procedures were carried out according to the guidelines of the Ethics committee, Medical University of Vienna and were approved by Federal Ministry of Education, Science and Culture, Austria (BMWFW-66.009/0114-WF/II/3b/2014). All efforts were made to minimize animal suffering and to reduce the number of animals used.

#### 1.2 Apparatus

The maze was made out of black plastic and kept at an elevation of 80 cm above the floor in a room with numerous visual cues. The central platform had a diameter of 50 cm with 12 arms (12cm x 60cm) projecting radially outwards. A plastic cylinder was used to restrict the movement of rats in the centre before the start of training. Lifting of the cylinder was controlled by a pulley system from the far end of the room.

#### 1.3 Procedure

RAM training was performed as described in Levin et al and Timofeeva et al **[15]** with some modification. In brief, rats were handled for 5 days for adaptation (30 min/day/rat) and to reduce the body weight to 85%. Water was provided ad libitum during the training. The amount of food (sniff Spezialdiäten GmbH, Germany) was provided to maintain a lean, healthy body weight of approximately 85% of the free-feeding weight during training. Out of 12 arms, eight arms were baited with food during the training and four remained un-baited. Before the start of the training, rats were given two habituation sessions in which food was placed all over the maze and rats were allowed to explore the maze and eat the food for five minutes. During the training session, the same arms were baited for each rat once at the beginning of each session to assess working memory, while the other four arms were always left un-baited in order to test reference memory. The pattern of baited and un-baited arms were consistent throughout testing for each rat but differed among rats. Each trial started by placing the rat onto the central platform, after 10 seconds the cylinder was lifted slowly and the rat was allowed to enter any arm. The session lasted eight minutes or until all eight baited arms were entered-whatever occurred first. Arms were baited only once and a repeated entry into a baited arm was counted as a working memory error (WME), whereas any entry into an un-baited arm was recorded as a reference memory error (RME). The rats were given 10 training sessions, one training per day. The training sessions were recorded with a computerized tracking video camcorder: 1/3 SSAM HR EX VIEW HAD. Six hours after the end of the tenth training animals were perfused using 1.5% paraformaldehyde for immunohistochemistry.

Untrained animals spent the same time in the RAM as their counterparts but the arms were not baited.

### 2. Immunofluorescence (IF) studies

#### 2.1 Animal perfusion and brain preparation

Eight mice each from the three groups were anesthetized with 400 mg/kg body weight of ketamine and 30 mg/kg body weight xylazine and perfused intracardially with ice-cold PBS (0.1 M) at a pH of 7.6 for approximately 2 min. The animals were perfused with 1.5% paraformaldehyde (PFA) in 0.1 M sodium phosphate buffer, whole brains were dissected and post-fixed for 1 h in the same 1.5% PFA, transferred to 0.1 M sodium phosphate buffer and 200 μm sections sliced with a vibrotome on the following day.

#### 2.2 Dendrite morphology/morphometry studies

DIL dye staining was performed on 2–3 sections of each animal. Solid DIL crystals(1,1´-dioctadecyl-3,3,3´,3´tetramethylindocarbocyanine perchlorate; Sigma, C D-282) were ground into a fine powder and applied to the slices with a Hamilton syringe (Hamilton, Switzerland)**[16]**. DIL crystals that adhered to the surface of the syringe tip were transferred to the surfaces of the slices. Special care was taken to avoid damaging the slices and to prevent the formation of dye clumps. Slices were incubated in 0.1 M sodium phosphate buffer at room temperature for 16 h to allow the DIL crystals to diffuse along the membranes. After 16 h slices were post-fixed with 1.5% PFA in 0.1 M sodium phosphate buffer for 30 min. All slices were counterstained for cell nuclei with DAPI (1.5 μg/mL, 4’,6-diamidino-2-phenylindole, Invitrogen, Carlsbad, CA, USA) in the final step of incubation. The slices were washed with 0.1 M sodium phosphate buffer for 2 min and mounted with fluorescent mounting media (Dako Fluorescent Mounting Media; S3023), and the spines were visualized.

Dendrites in CA1 area (apical and basal dendrites), CA3 area (apical and basal dendrites) and also in the molecular layer of dentate gyrus, dendrites were randomly selected for imaging DIL fluorescence. Primary dendrites were excluded from the analysis. Dendritic spines were imaged with a Zeiss 700 confocal laser scanning microscope (LSM 700). Dendrites were randomly imaged using an oil immersion 63× objectives at 2 × zoom and 1024 × 1024 pixel resolution. The spectral detectors were adjusted to capture emission from a helium/neon laser at wavelengths of 555–630 nm for DIL staining, and the pinhole diameter was maintained at 1 Airy unit. The Z stack acquisitions were performed with fluorescence z-stacks of 5–8 μm thickness consisting of sections at 0.3 μm increments were rapidly scanned within a 33.8 × 33.8 μm imaging area. The image acquisition was set at a range of 8 bits. Spine densities were calculated by quantifying the number of spines per 30 μm of dendritic length. For each group approximately 40–50 apical and basal dendrites each from CA1 and CA3 and40-50 dendrites from molecular layer of dentate gyrus were quantified separately. Dendritic length was measured using NIH Image J software and spine numbers were counted manually in 3 dimensions using Z stacks. Spine type analyses were performed using the NeuronStudio software (http://research.mssm.edu/cnic/tools-ns.html) **[17–19]**. Spine shapes were classified as thin, mushroom, stubby and others by “NeuronStudio” on the basis of the aspect ratio, head-to-neck ratio and head diameter. Spines with a neck can be classified as either thin or mushroom and those without a neck are classified as stubby. Spines with a neck are labeled as thin or mushroom on the basis of head diameter. This method is in agreement with well-accepted methods for spine type classification **[20, 21]**. The analysis was performed blinded.

### 3. Statistical analyses

Statistical analyses were performed with Graph Pad software version 5.00.288 (Prism; Graph Pad, San Diego, CA, USA). For RAM, data was analyzed by repeated measures one-way ANOVA followed by Bonferroni post-hoc test. The data are reported as means ± SEMs. Spine density was calculated by dividing total number of spines per length of dendrite. For spine types, the number of each spine type relative to total number of spines was calculated. For analyzing group mean differences in spine densities and types of spines, multi-comparison one-way ANOVA followed by Bonferroni post-hoc test was used. The data are reported as means ± SDs. Spearman Correlation was used for correlation analysis between spine density and WME and latency.

## Results

### Rats show improved performance in the RAM

**Fig 1:**
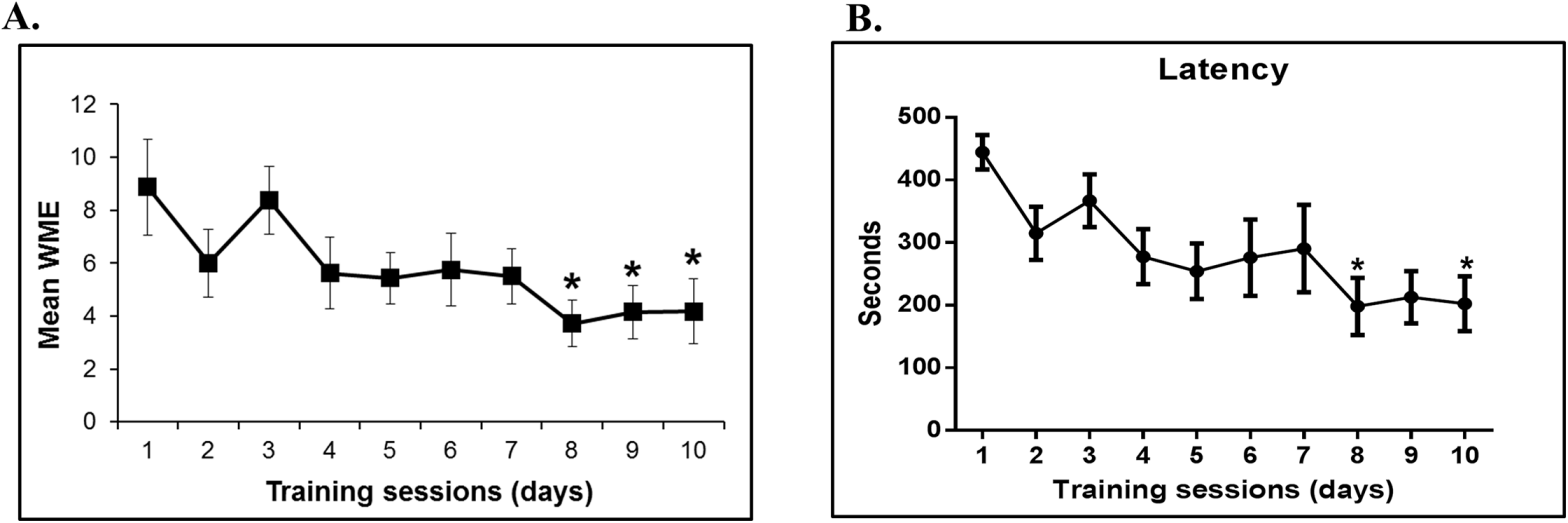
Radial arm maze training. (A) Working memory error curve (WME); (B) Latency curve. WME were significantly decreased on day- 8, 9 and 10 compared to day-1. Mean and SEM are shown in the graph. Latency was significantly decreased on day- 8 and day- 10 compared to day- 1. The total numbers of errors were analyzed using repeated measurements ANOVA (* P < 0.05, F = 3.437)]

Rats were trained in the RAM analyzing working memory error (WME) and latency revealing that rats showed improved performance in the RAM task. Significant reduction of WMEs **(P = 0.0018, f value = 3.437)** was observed over the course of trainingwhen rats **(n = 8)** were trained in the twelve arm radial maze. Fig 1A shows the trend of decreasing number of WMEs made by the animals with respect to the training sessions. WMEs became statistically different from day 8 onwards.

The RMEs counted were not significant and representative fig is provided in Fig A in S1 Fig. Significant reduction of latency **(P = 0.0101, f value = 2.738, n = 8)** was observed as provided in Fig 1B. Latency was significant on day 8 **(P = 0.0294)** and day 10 **(P = 0.0383)**.

The untrained animals **(n = 8)** were placed in the RAM for exactly the same time as that of the corresponding trained animals with the same protocol except that the arms were not baited. Thus, they were exploring the RAM randomly without any particular aim (in this case, searching food pellets). The cage control animals **(n = 8)** remained in their homecage throughout the experiments.

Trained, untrained and cage control rats **(n = 8 per group)** were perfused after 6h transcardially with 1.5% paraformaldehyde (PFA) in 0.1M sodium phosphate buffer, whole brains were dissected and 200 μm sections were sliced with a vibrotome on the following day. These slices were stained by using DIL dye staining and were examined by LSM 700 microscope in three different areas of the brain hippocampus: CA1 (apical and basal) dendrites, CA3 (apical and basal) dendrites and molecular layer of dentate gyrus. In case of CA1 and CA3, care was taken to choose pyramidal neurons, which were well separated from each other. Representative DIL dye staining in CA1 is provided in Fig B in S1 Fig. Subsequently, dendritic spine density was examined.

### Spine density of apical and basal dendrites in the CA1 area of hippocampus was increased following training in the RAM

Totally 40 apical and basal dendrites each were selected from each rat (n = 8) (5 neurons per each rat) per each group (trained, untrained and cage control). The total spines studied for each group were: for cage control, 3613 spines from apical and 3752 from basal; for untrained, 3742 spines from apical and 3363 spines from basal; while for the trained group, 5647 spines from apical and 6176 from the basal were counted.

**Fig 2:**
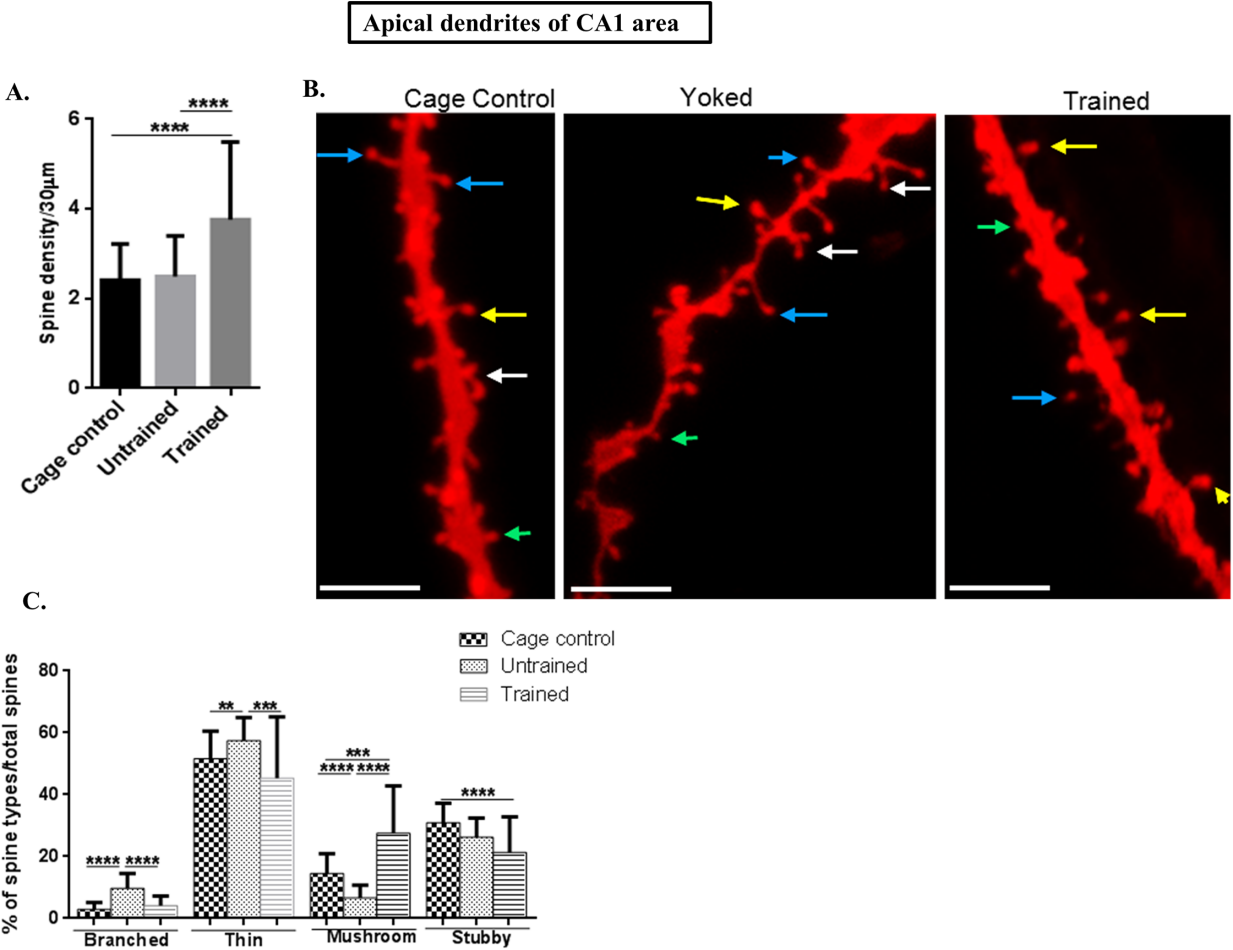
Spine morphology of CA1 apical dendrites. (A) Graphical representation of spine density/30μm of apical dendrites in the CA1 subarea; (B) Representative images of DIL dye staining of apical dendrites of the CA1 subarea where the mushroom spines are represented by yellow colour, thin spines by blue colour, branched spines by white colour and stubby spines by green colour (scale bar = 20 μm); (C) Graphical representation of percentage of branched, thin, mushroom and stubby spines per total spines of apical dendrites of CA1 subarea.

**Fig 3:**
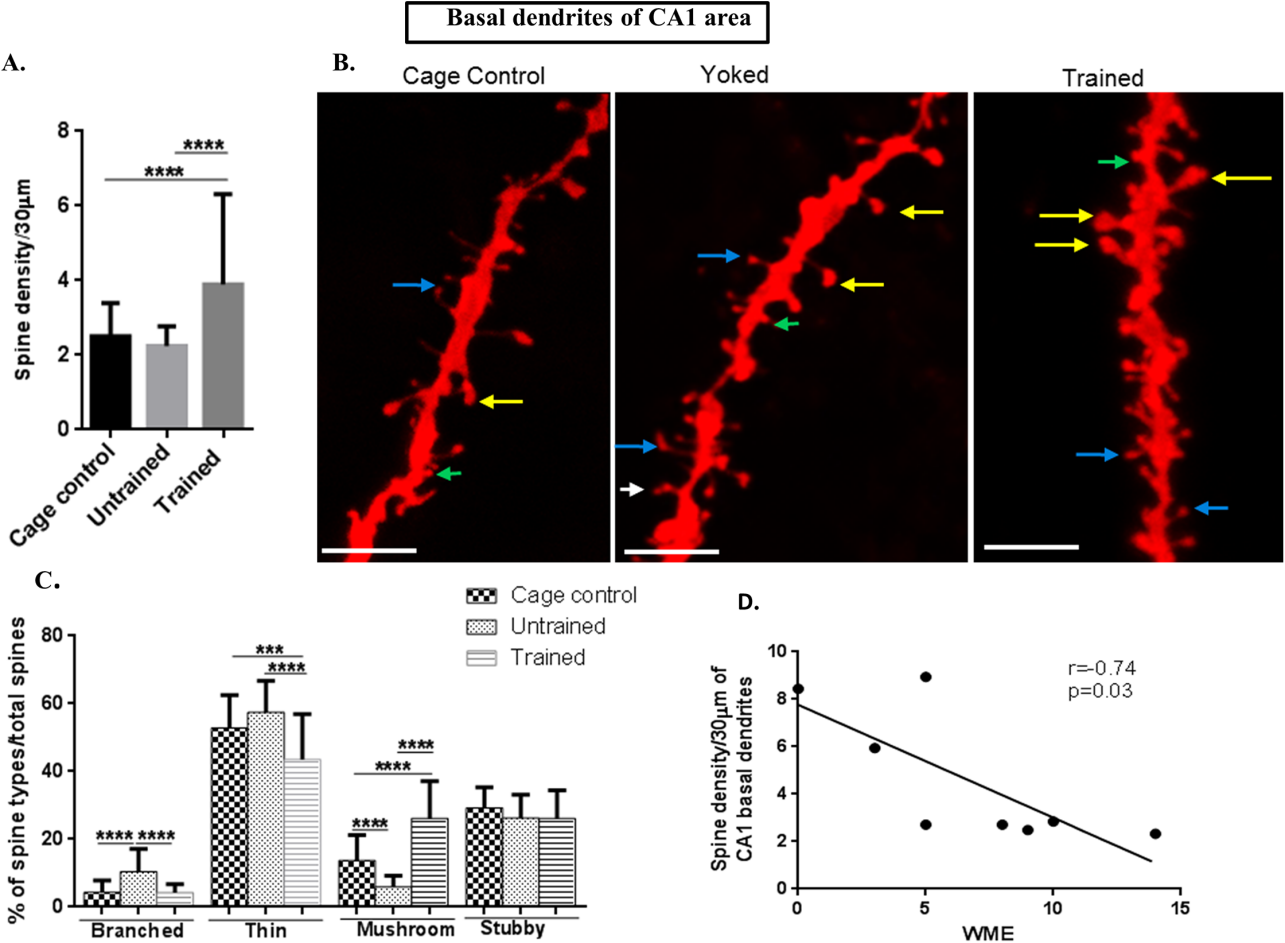
Spine morphology of CA1 basal dendrites. (A) Graphical representation of spine density/30μm of basal dendrites in the CA1 subarea; (B) Representative images of DIL dye staining of basal dendrites of the CA1 subarea (scale bar = 20 μm); (C) Graphical representation of the percentage of branched, thin, mushroom and stubby spines per total spines of basal dendrites of the CA1 subarea; Spine density and mushroom spines were increased in the trained group in the CA1 apical and basal dendrites; (D) Negative Spearman Correlation of spine density/30μm of basal dendrites of CA1 subarea with working memory error (WME) (r = -0.74, p = 0.04; r ranges from -1 to 0 to +1 where, -1 = 100% negative correlation, 0 = no correlation and +1 = 100% positive correlation). Statistical evaluation was carried out by multi-comparison one-way ANOVA followed by post-hoc Bonferroni's test. Data is provided as mean±SD (*P < 0.05;**P ≤ 0.01 ***P < 0.001; ****P < 0.0001)].

CA1 apical **(f value = 19.42, P value 0.0001)** and basal **(f value = 16.93, P value 0.0001)** dendritic spine densities were increased in the trained group in comparison to both, untrained and cage control groups as shown in Figs 2A and 3A, respectively.

### Mushroom spines were increased on CA1 apical as well as basal dendrites following RAM performance

Spine morphology was assessed following RAM performance wherein the individual spines types as branched, mushroom, thin and stubby were analyzed.

The percentage of branched spines in apical **(n = 40, f value = 53.78, P value ˂ 0.0001)** and basal dendrites **(n = 40, f value = 29.95, P value ˂ 0.0001)** in CA1, as shown in Fig 2B and 2C (for apical dendrites); Fig 3B and 3C (for basal dendrites) showed that branching of spines was significantly decreased in trained and cage control groups in comparison to untrained animals.

The percentage of thin spines on both apical **(f value = 9.25, p value = 0,0002)** as well as basal dendrites **(f value = 20.75, P value ˂ 0.0001)** in CA1 area was decreased in trained animals in comparison to untrained and cage controls in the same area as shown in Fig 2B and 2C (for apical dendrites) and Fig 3B and 3C (for the basal dendrites).

Herein, it was shown that the percentage of mushroom spine types of apical **(f value = 51.39, P value ˂ 0.0001)** as well as basal dendrites **(f value = 83.94, P value ˂ 0.0001)** was significantly increased in the trained group in comparison to the untrained and cage controls as shown in Fig 2B and 2C (for apical dendrites) and Fig 3B and 3C (for the basal dendrites).

The percentage of stubby spines was decreased in the trained group in comparison to the untrained group and the cage control in CA1 apical dendrites **(f value = 14.6, P value ˂ 0.0001)** and for the basal dendrites **(f value = 3.04, Pvalue = 0.0508)**. The dynamics of stubby spines is not well understood but they are mainly considered to be immature spines [22]. The representative images of spine density and types of CA1 are shown in Fig 2B and 2C (for apical dendrites) and Fig 3B and 3C (for the basal dendrites).

Although spine morphology and counts have been already carried out in a learning and memory paradigm, the Morris Water Maze, no direct link between learning and memory and spine counts and morphology was reported and therefore the corresponding correlations were performed herein [3].

### CA1 basal dendritic spines are negatively correlated with WMEs

In order to establish a link between dendritic spine density and the training in RAM, spine density was correlated with the corresponding WMEs and latency. Spines on CA1 apical dendrites were not correlated, however, spine density of basal dendrites was negatively correlated with corresponding WMEs (**r = -0.74, p value = 0.03)** as shown in Fig 3D.

### Spine density was increased in apical and basal dendrites of CA3 following RAM performance

There is no report on spine numbers or morphology following training in the RAM in CA3 and dentate gyrus in spatial working memory, so the study was performed in the RAM.

In the CA3 area totally 40 apical and basal dendrites each were selected from each rat (n = 8) (5 neurons per each rat) per group (trained, untrained and cage control). The total spines studied for each group were: for cage control, 3739 spines from apical and 3575 from basal; for untrained, 3843 spines from apical and 3898 spines from basal; while for the trained group, 7390 spines from apical and 4685 from the basal were counted.

**Fig 4:**
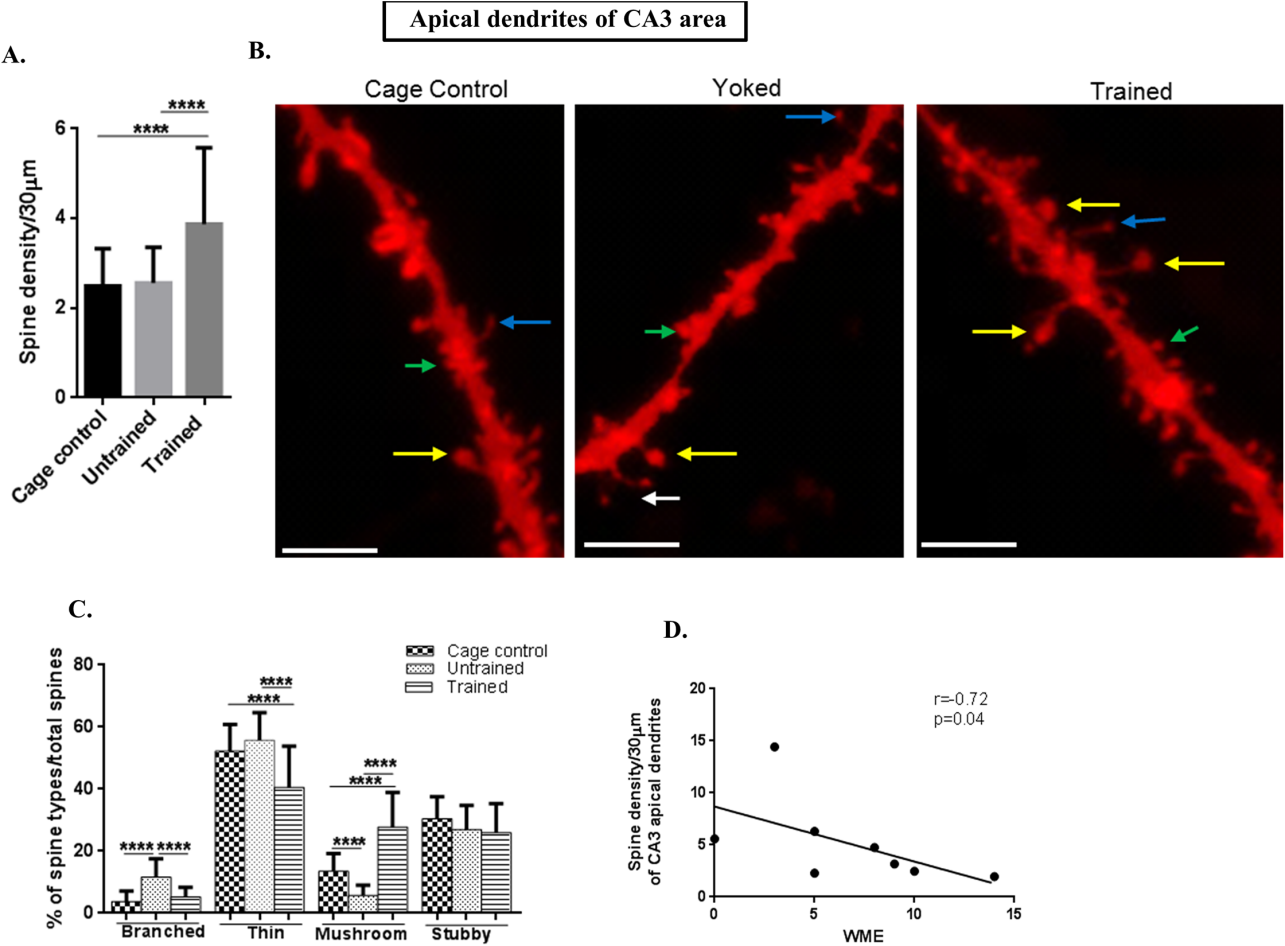
Spine morphology of CA3 apical dendrites. (A) Graphical representation of spine density/30μm of apical dendrites of CA3 subarea; (B) Representative images of DIL dye staining of apical dendrites of CA3 subareawhere the mushroom spines are represented by yellow colour, thin spines by blue colour, branched spines by white colour and stubby spines by green colour; (scale bar = 20 μm); (C) Graphical representation of percentage of branched, thin, mushroom and stubby spines per total spines of apical dendrites of CA3 subarea;(D) Negative Spearman Correlation of spine density/30μm of apical dendrites of CA3 subarea with working memory error (WME) (r = -0.72, p = 0.04; r ranges from -1 to 0 to +1 where -1 = 100% negative correlation, 0 = no correlation and +1 = 100% positive correlation).

**Fig 5:**
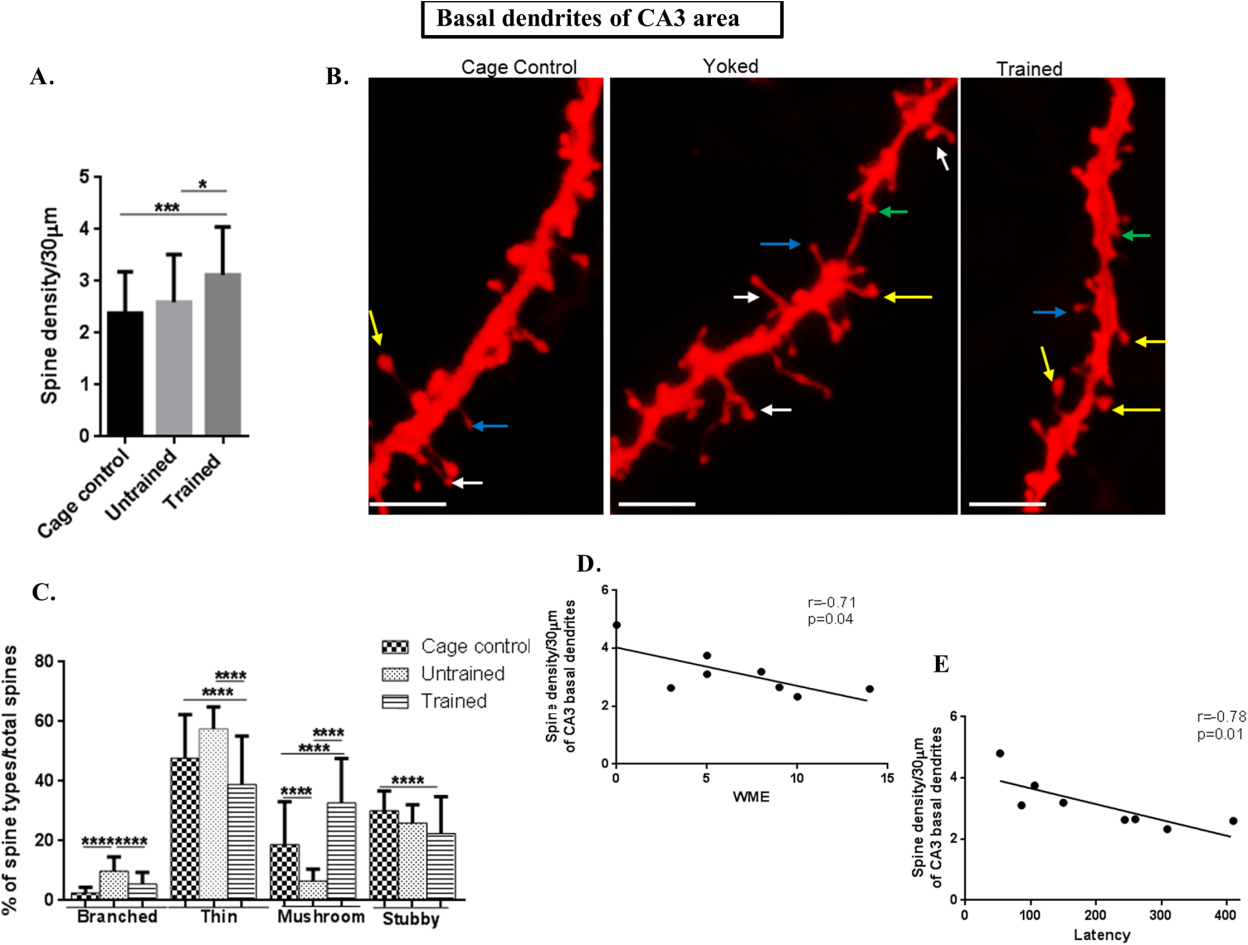
Spine morphology of CA3 basal dendrites. (A) Graphical representation of spine density/30μm of basal dendrites of CA3 subarea; (B) Representative images of DIL dye staining of basal dendrites of CA3 subarea (scale bar = 20 μm);(C) Graphical representation of percentage of branched, thin, mushroom and stubby spines per total spines of basal dendrites of CA3 subarea; (D) Negative Spearman Correlation of spine density/30μm of basal dendrites of CA3 subarea with working memory error (WME) (r = -0.72, p = 0.04); (E) Negative Spearman correlation of spine density/30 μm of basal dendrites of CA3 subarea with latency (r = -0.78, p = 0.01, r ranges from -1 to 0 to +1 where -1 = 100% negative correlation, 0 = no correlation and +1 = 100% positive correlation); (J) Negative Spearman Correlation of spine density/30μm of basal dendrites of CA3 subarea with reference memory error (r = -0.71, p = 0.04); Statistical evaluation was carried out by multi-comparison one-way ANOVA followed by post-hoc Bonferroni's test. Data is provided as mean±SD (*P < 0.05;**P ≤ 0.01 ***P < 0.001; ****P < 0.0001)].

CA3 apical dendritic spine density was also increased in the trained group **(f value = 30.33, P value ˂ 0.0001)** and in CA3 basal dendrites **(f value = 9.331, P value = 0.0002)** in comparison to both, untrained and cage control groups, as shown in Figs 4A and 5A.

In dentate gyrus, the molecular layer was selected and totally 40 dendrites were selected from each rat (n = 8) (5 neurons per each rat) per each group (trained, untrained and cage control). The total spines studied for each group were: for cage control, 3681 spines; for untrained, 4139 spines and for trained group, 5134 spines.

**Fig 6:**
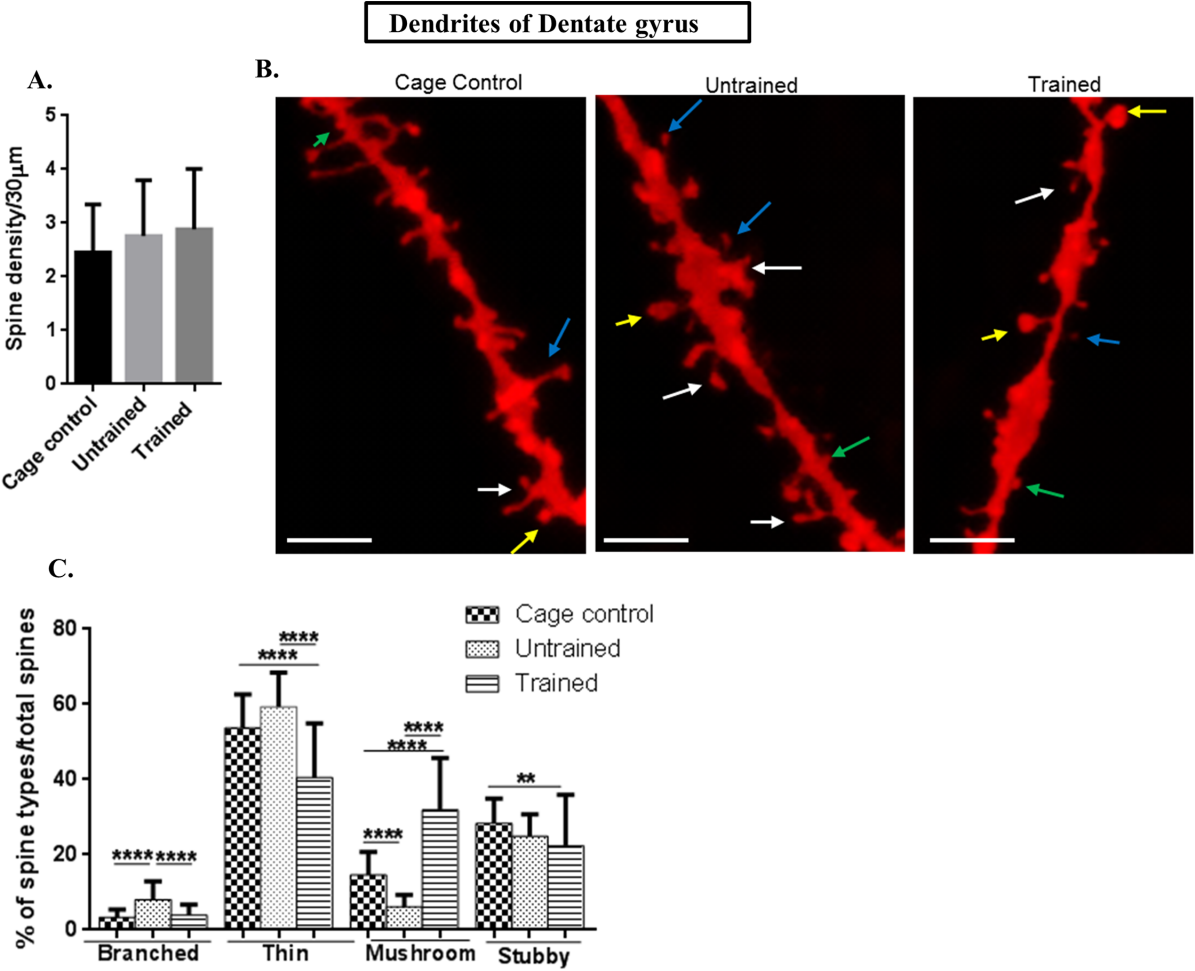
Spine morphology of molecular layer of dentate gyrus. (A) Graphical representation of spine density/30μm of molecular layer of dentate gyrus; (B) Representative images of DIL dye staining of of molecular layer of dentate gyrussubarea where the mushroom spines are represented by yellow colour, thin spines by blue colour, branched spines by white colour and stubby spines by green colour (scale bar = 20 μm); (C) Graphical representation of percentage of branched, thin, mushroom and stubby spines per total spines of molecular layer of dentate gyrus; Statistical evaluation was carried out by multi-comparison one-way ANOVA followed by post-hoc Bonferroni's test. Data is provided as mean±SD (*P < 0.05;**P ≤ 0.01 ***P < 0.001; ****P < 0.0001).]

Dendritic spine density in the dentate gyrus **(f value = 2.275, P value = 0.1065)** was comparable to trained and cage control groups as shown in Fig 6A.

### Spine morphology in CA3 and dentate gyrus showed same trend as that of CA1 area

The branched spines in apical**(f value = 45.01, P value ˂ 0.0001)** and basal dendrites**(f value = 43.36, P value ˂ 0.0001)** of CA3 and dentate gyrus**(f value = 24.01, P value = ˂ 0.0001)** showed significantly decreased numbers in trained and cage control in comparison to that of untrained animals. The graphical figs for CA3 are illustrated in Figs 4C (apical) and 5C (basal), and in the dentate gyrus it is shown in Fig 6C.

The percentage of thin spines on both, apical as well as basal dendrites of CA3 and dentate gyrus was decreased in trained in comparison to untrained and cage controls. In the untrained group the percentage of thin spines in the apical part of CA3 was **(f value = 28.51, P value ˂ 0.0001)**, while in the basal part it was **(f value = 31.59, P value ˂ 0.0001)** as shown in Figs 4C and 5C, respectively and in the dentate gyrus it was **(f value = 37.19, P value ˂ 0.0001)** as given in Fig 6C.

The percentage of mushroom spine types of apical **(f value = 110, P value ˂ 0.0001)** as well as basal dendrites (**f value = 90.24, P value ˂ 0.0001)** of CA3 and dentate gyrus**(f value = 107, P value ˂ 0.0001)** were significantly increased in the trained group in comparison to the untrained and cage controls as shown in Figs 4C (CA3 apical), 5C (CA3 basal) and 6C for dentate gyrus.

The percentage of stubby spines were decreased in the trained and untrained groups in comparison to the cage control in CA3 apical dendrites **(f value = 4.098, P value = 0. 185)** and in a basal CA3 area **(f value = 9. 363, P value = 0.0001**) and in the dentate gyrus (**f value = 4.995, P value = 0. 008)** as shown in Figs 4C (for apical CA3 dendrites), 5C (for basal CA3 dendrites) and 6C (for dentate gyrus dendrites).

Thus, CA3 and dentate gyrus show the same trend as that of the CA1 area where the number of mushroom spines was increased following training.

### CA3 basal dendritic spines were negatively correlated to WME and latency

In order to establish a link between dendritic spine density and the training in RAM, spine density was correlated with the WME and latency. CA3 apical **(r = -0.72, p = 0.04, n = 8)** and basal **(r = -0.71, p = 0.04,n = 8)** dendritic spines were negatively correlated with WMEs as shown in Figs 4D and 5D. In addition, the basal spine density in CA3 **(r = -0.78, p = 0.01, n = 8)** was negatively correlated with corresponding latency as shown in Fig 5E. The corresponding correlations indicate that spine densities of basal dendrites are associated with RAM performance.

## Discussion

The increase in spine density on pyramidal neurons indicates an increase of excitatory synapses as it was previously reported that an increase in the spine density refers to increase in number of excitatory synapses per neuron associated with the task **[20, 21]**. Although it has been shown that spine density following spatial learning task was significantly increased in CA1 **[20, 21]**, there are no published data showing increased spine density after a performance in the RAM during memory encoding. It was observed that spine density was increased six hours following RAM task in CA1 and the CA3 sub area of the hippocampus. The RAM is a complex memory paradigm comprising many components as working memory, spatial memory and long-term memory. Involvement of the hippocampus in working memory has been reported [23], however, increased dendritic complexity following working memory in the hippocampus is reported in CA1 where only spine count was analyzed showing increased spine density following consolidation of working memory [24].

In this context the use of the RAM could offer advantages as the increase of synapse is not only following spatial memory but following an intricate paradigm as it is more commonly used to check memory performance for testing spatial working memory and spatial reference error. The observed increase of excitatory synapses in CA1 and CA3 may have resulted in increased synaptic plasticity that in turn represents the basis for learning and memory mechanisms during the acquisition of complex memory in RAM performance.

The numbers of dendritic spines increase in both, apical and basal dendrites in CA1 and CA3 sub area suggests that these synapses show equal propensity for RAM performance as it was previously shown that the basal and apical dendritic synapses showed equal propensity for an input-specific LTP **[25]**.

Traditionally, spine morphology was mainly analyzed using the Golgi-Cox method which has several limitations. The Golgi method provides uneven, low frequency staining, which consequences in the uncertainty of a selection bias. Thus use of florescent marker as DIL staining overcomes these limitations and makes the technique more reliable [26].

The spine density of CA1 and CA3 and corresponding correlations between WMEs established a link between spine density and working memory. In addition, corresponding correlations with latency and CA3 reveals direct significance between memory performance and spine count.

The spine morphology analysis revealed that CA1, CA3 and dentate gyrus showed the same trend of Results for different spine types. Although spine morphology and spine dynamics is studied, the mechanism behind some spine types is still unknown. It is proposed that dendritic spines are modified within minutes that may correspond to the consolidation of initial memories, whereas increased dendritic spine density after longer periods of time may be part of a type of continued plasticity necessary for storage of long term memories [27]. It is shown that acquisition of spatial task showed transient synaptic changes in the CA1 area [28], however, the spine morphology followed after acquisition of memory following RAM performance is not studied.

It was demonstrated that branched spines were increased in the untrained animals on apical and basal dendrites of CA1 and CA3 and on molecular layer of dentate gyrus. The dendritic branching or splitting of spines with two heads on the same presynaptic button is proposed to be essential for increased synaptic plasticity in LTP but the underlying mechanism is still elusive**[29]**. On contrary, branching was lower in trained than in untrained animals, i.e. at memory acquisition, which is not a contradiction as branching was studied in a spatial memory paradigm and no LTP was measured **[30]** and, moreover, the branching mechanism is not known.

It was demonstrated that number of thin spines were decreased on CA1, CA3 and dentate gyrus dendrites. Thin spines are proposed to be transient spines, that are mainly known as ‘learning spines’ and are mainly increased during the acquisition of the task **[22]**. The RAM performance task consists of ten days training and on day 10 acquisition of memory was assessed. However number of thin spines where decreased in the trained animals doesn’t contradicts the results as still there there is no direct evidence to support this idea [31]. This future findings are needed to study spine dynamics.

The increase in mushroom spines (which have been proposed to be “memory spines” **[22]** in the CA1,CA3 and dentate gyrus, may complement information on spine numbers and verify that mice memorized and acquired the task. The mushroom spines are known to have larger postsynaptic densities and were proposed to recruit more α-Amino-3-hydroxy-5-methyl-4-isoxazolepropionic acid (AMPA) receptors [32].

Stubby spines were increased in cage control in studied areas of hippocampus, however, the dynamics of stubby spines is not well understood but they are mainly considered to be immature spines [22].

The changes of spine types in CA3 and dentate gyrus is confirmatory as it is known that the dentate gyrus plays a significant role in RAM performance **[33],[34]**. Moreover, lesions of dentate gyrus and CA3 have shown to impair spatial memory tasks **[35]**. The changes in spine morphology in sub areas of hippocampus may represent activation of the tri-synpatic loop.

## Conclusion

Taken together, the results demonstrate that spine density was increased paralleling RAM performance and increased excitatory synapses were observed during spatial memory formation. The increase in mushroom spines may confirm that mushroom spines are "memory spines". The spine density increase in the individual areas of the hippocampus may point to importance of these areas in the RAM performance task. In addition to previous work it was shown that increased spine density is not only following spatial learning but is directly linked to performance (working memory errors and latency) in the RAM.

The method may be used as a morphological correlate for learning and memory studies and may be even used for evaluation of cognitive enhancement in neuropharmacological settings.

## Supporting Information

S1 Fig (A) Reference memory error (RME) curve and (B) DIL dye staining image. (TIF)

S1 Table Statistical analysis of spine density. (DOCX)

S2 Table Statistical analysis of types of spine of CA1 sub area. (DOCX)

S3 Table Statistical analysis of types of spine of CA3 sub area. (DOCX)

S4 Table Statistical analysis of types of spine of dentate gyrus. (DOCX)
