# Supplementary figures and images for: Spatial and Working Memory Is Linked to Spine Density and Mushroom Spines
Source: PLoS One. 2015 Oct 15;10(10):e0139739. doi: 10.1371/journal.pone.0139739 (PMC4607435; doi:10.1371/journal.pone.0139739)

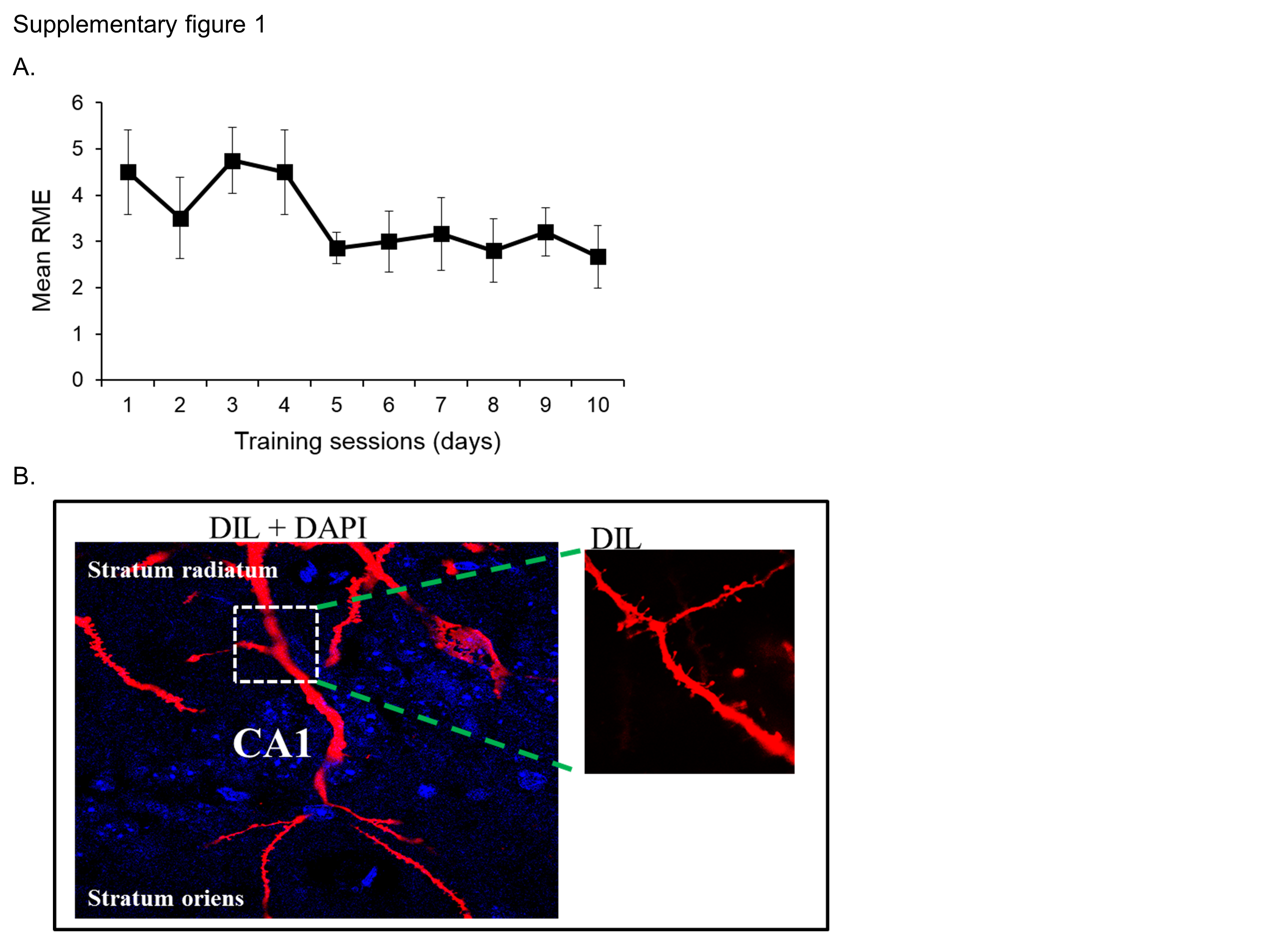

Supplement: S1 Fig — (TIF) [file pone.0139739.s001.tif]
