# Supplementary material for: Spatial and Working Memory Is Linked to Spine Density and Mushroom Spines
Source: PLoS One. 2015 Oct 15;10(10):e0139739. doi: 10.1371/journal.pone.0139739 (PMC4607435; doi:10.1371/journal.pone.0139739)
Supplement: S1 Table — (DOCX) [file pone.0139739.s002.docx]

**Supplementary table 1**

**Statistical analysis of spine density**

| **Hippocampal sub area** | **Spine density (Mean ± Standard deviation)** | | | **F value** | **P value** |
| --- | --- | --- | --- | --- | --- |
|  | **Cage control** | **Untrained** | **Trained** |  |  |
| CA1 (apical) | 2.408 ± 0.8081 | 2.408 ± 0.8081 | 3.765 ± 1.73 | 19.42 | < 0.0001 |
| CA1 (basal) | 2.501 ± 0.8863 | 2.242 ± 0.5371 | 3.891 ± 2.42 | 16.93 | < 0.0001 |
| CA3 (apical) | 2.493 ± 0.8358 | 2.562 ± 0.7889 | 3.87 ± 1.708 | 30.33 | < 0.0001 |
| CA3 (basal) | 2.383 ± 0.8033 | 2.599 ± 0.9152 | 3.123 ± 0.9206 | 9.331 | 0.0002 |
| Dentate gyrus | 2.454 ± 0.89 | 2.759 ± 1.034 | 2.883 ± 1.122 | 2.275 | 0.1065 |
