# Supplementary material for: Spatial and Working Memory Is Linked to Spine Density and Mushroom Spines
Source: PLoS One. 2015 Oct 15;10(10):e0139739. doi: 10.1371/journal.pone.0139739 (PMC4607435; doi:10.1371/journal.pone.0139739)
Supplement: S2 Table — (DOCX) [file pone.0139739.s003.docx]

**Supplementary table 2**

**Statistical analysis of types of spines of CA1 subarea**

| **Types of spines** | **CA1 apical (Mean ± SEM)** | | | **P value** | **F value** |
| --- | --- | --- | --- | --- | --- |
|  | **Cage control** | **Untrained** | **Trained** |  |  |
| Branched | 2.986 ± 0.32 | 9.674 ± 0.73 | 3.731 ± 0.73 | < 0.0001 | 53.78 |
| Thin | 51.63 ± 1.33 | 57.44 ± 1.12 | 45.39 ± 3.01 | 0.0002 | 9.25 |
| Mushroom | 14.51 ± 0.95 | 6.643 ± 0.61 | 27.53 ± 2.28 | < 0.0001 | 51.39 |
| Stubby | 30.88 ± 0.95 | 26.24 ± 0.92 | 21.33 ± 1.712 | < 0.0001 | 14.6 |
|  | **CA1 basal (Mean ± SEM)** | | |  |  |
|  | **Cage control** | **Untrained** | **Trained** |  |  |
| Branched | 4.272 ± 0.50 | 10.46 ± 0.95 | 4.223 ± 0.35 | < 0.0001 | 29.95 |
| Thin | 52.81 ± 1.37 | 57.4 ± 1.32 | 43.52 ± 1.89 | < 0.0001 | 20.75 |
| Mushroom | 13.71 ± 1.05 | 5.934 ± 0.46 | 26.17 ± 1.54 | < 0.0001 | 83.94 |
| Stubby | 29.21 ± 0.85 | 26.2 ± 0.97 | 26.09 ± 1.17 | 0.0508 | 3.04 |
