# Supplementary material for: Spatial and Working Memory Is Linked to Spine Density and Mushroom Spines
Source: PLoS One. 2015 Oct 15;10(10):e0139739. doi: 10.1371/journal.pone.0139739 (PMC4607435; doi:10.1371/journal.pone.0139739)
Supplement: S3 Table — (DOCX) [file pone.0139739.s004.docx]

**Supplementary table 3**

**Statistical analysis of types of spines of CA3 subarea**

| Types of spines | CA3 apical (Mean ± SEM) | | | P value | F value |
| --- | --- | --- | --- | --- | --- |
|  | Cage control | Untrained | Trained |  |  |
| Branched | 3.798 ± 0.4689 | 11.62 ± 0.8442 | 5.737 ± 0.4639 | < 0.0001 | 45.01 |
| Thin | 52.25 ± 1.218 | 55.68 ± 1.262 | 40.55 ± 1.882 | < 0.0001 | 28.51 |
| Mushroom | 13.52 ± 0.8082 | 5.765 ± 0.4606 | 27.73 ± 1.588 | < 0.0001 | 110 |
| Stubby | 30.43 ± 1.01 | 26.94 ± 1.107 | 25.98 ± 1.332 | 0.0185 | 4.098 |
|  | CA3 basal (Mean ± SEM) | | |  |  |
|  | Cage control | Untrained | Trained |  |  |
| Branched | 2.569 ± 0.2881 | 9.866 ± 0.6768 | 5.483 ± 0.5912 | < 0.0001 | 43.36 |
| Thin | 50.91 ± 1.489 | 57.55 ± 1.037 | 38.89 ± 2.303 | < 0.0001 | 31.59 |
| Mushroom | 15.06 ± 1.084 | 6.584 ± 0.5533 | 32.73 ± 2.108 | < 0.0001 | 90.24 |
| Stubby | 30.08 ± 0.94 | 26 ± 0.8607 | 22.44 ± 1.75 | 0.0001 | 9.363 |
