# Supplementary material for: Spatial and Working Memory Is Linked to Spine Density and Mushroom Spines
Source: PLoS One. 2015 Oct 15;10(10):e0139739. doi: 10.1371/journal.pone.0139739 (PMC4607435; doi:10.1371/journal.pone.0139739)
Supplement: S4 Table — (DOCX) [file pone.0139739.s005.docx]

**Supplementary table 4**

**Statistical analysis of types of spines of dentate gyrus**

| Types of spines | Dentate gyrus (Mean ± SEM) | | | P value | F value |
| --- | --- | --- | --- | --- | --- |
|  | Cage control | Untrained | Trained |  |  |
| Branched | 3.388 ± 0.2966 | 8.004 ± 0.7449 | 3.985 ± 0.4266 | < 0.0001 | 24.01 |
| Thin | 53.69 ± 1.274 | 59.34 ± 1.298 | 40.49 ± 2.059 | < 0.0001 | 37.19 |
| Mushroom | 14.62 ± 14.62 | 6.067 ± 0.4587 | 31.92 ± 1.974 | < 0.0001 | 107 |
| Stubby | 28.31 ± 0.9547 | 24.87 ± 0.8425 | 22.33 ± 1.945 | 0.008 | 4.995 |
